# Using care and support planning to implement routine falls prevention and management for people living with frailty: A qualitative evaluation

**DOI:** 10.1371/journal.pone.0275974

**Published:** 2022-10-11

**Authors:** Tracy Finch, Michaela Fay, Joanne Smith, Helen Kleiser, Deborah Dews, Sue Roberts, Fiona Shaw, Shona Haining, Lindsay Oliver

**Affiliations:** 1 Department of Nursing, Midwifery & Health, Northumbria University, Newcastle upon Tyne, United Kingdom; 2 North of England Commissioning Support, Newcastle upon Tyne, United Kingdom; 3 Year of Care Partnerships, Northumbria Healthcare NHS Foundation Trust, Wansbeck Hospital, Ashington, Northumberland, United Kingdom; 4 Rowlands Gill Medical Centre, Rowlands Gill, Tyne and Wear, United Kingdom; 5 Campus for Ageing and Vitality, Newcastle upon Tyne Hospitals NHS Foundation Trust, Newcastle upon Tyne, United Kingdom; University of Edinburgh, UNITED KINGDOM

## Abstract

**Background:**

Frailty is a key issue in current healthcare delivery and falls is an important component. Care and support planning (CSP) is an established approach to managing long term conditions (LTCs) and has potential to provide more person-centred care for those at risk of falling. This qualitative evaluation aimed to understand the barriers and success criteria involved in incorporating falls assessment and management into the CSP process.

**Methods:**

CSP for falls prevention was implemented in eight general practices in the North of England. Six of the eight practices participated in the qualitative evaluation. Seven group interviews were undertaken with staff (n = 31) that included practice nurses, health care assistants, nurses, and administrative staff (n = 2–8 per group). Observations of the falls and CSP training provided additional data. Interviews covered experiences and potential impacts of training, and processes of implementation of the programme, and were informed by normalisation process theory. Thematic analysis was undertaken using a team-based approach.

**Results:**

Although successfully implemented across the practices, how established CSP was and therefore ‘organisational readiness’ was an overarching theme that illustrated differences in how easily sites were able to implement the additional elements for frailty. The challenges, successes and impacts of implementation are demonstrated through this theme and four further themes: training resources and learning; positive impacts of the programme (including enabling easier conversations around ‘frailty’); integrating work processes/work with patients; and dealing with uncertainty and complexity.

**Conclusions:**

Care and Support Planning services designed to target frailty and falls is feasible and can successfully be delivered in the primary care setting, if key enablers are promoted and challenges to implementation addressed from planning through to integration in practice.

## Introduction

Frailty is a key issue in current healthcare delivery and falls is an important component. The number of people living with frailty is increasing year on year, placing an increasing burden on our health and social care systems [1].

Frailty, as a functional state, has the features of a long-term health condition [2]. characterised by loss of physical, emotional and cognitive resilience as a result of the accumulation of multiple health deficits [3]. An individual living with increasing frailty is more vulnerable to poorer health outcomes, even from a minor illness or stressor event.

One in three people over the age of 65 are reported to fall every year in England with those living with frailty at higher risk [4]. Contributing factors for falls include polypharmacy, nutritional compromise, alcohol intake, environmental hazards, and functional decline [5]. These may be modifiable through interventions such as medicine review, environmental assessment and participation in targeted exercise [6]. There is a significant body of research that supports strength and balance training in preventing falls and ameliorating frailty [4, 7]. This suggests that discussing and addressing potential risk factors as part of a preventative approach to supporting older adults to ‘age well’ could offer considerable benefits.

Care and Support Planning (CSP) [8] is a systematic approach to providing routine care for people with single and multiple long-term conditions. The process creates the space for a more meaningful conversation between a prepared person and a trained practitioner which brings together all the conditions or issues a person may live with including a preventive approach. It is forward looking, and solution focussed and brings together traditional clinical issues with support for self-management during day-to-day living, including the opportunity to link with supportive activities in the community (social prescribing). The aim of the CSP conversation is to ensure that what matters most to the person (patient) is discussed alongside any professional concerns that have been identified. The conversation has a particular style and flow with the practitioner taking on the role of ‘facilitator’ rather than ‘fixer’.

The CSP process thus provides an opportunity to test systematically whether a holistic approach to the prevention, early assessment and appropriate management of falls and frailty might be included as part of routine care in general practice [3].

In the UK, the Year of Care (YOC) Programme has over 15 years of experience of development and implementation of CSP in single and multiple long-term conditions including providing training and support to embed the organisational and cultural changes required [8]. CSP has become the standard approach to the management of long-term conditions in 59 out of 63 general practices in Newcastle Gateshead Clinical Commissioning Group (CCG) where the project was based. The aim was to test the feasibility of including frailty and falls identification and prevention within this process. This paper describes the qualitative arm of a mixed methods study [9] to understand the barriers and success criteria involved in incorporating falls assessment and management into the CSP process, and to make recommendations for future development.

## Methods

### The project context

The programme practices were recruited via the CCG network (bulletins and newsletters) and informal approaches to practices who were either interested in frailty, strong advocates of CSP or had already expressed interest in taking forward work around frailty and CSP. All practices had to have established CSP processes in place as judged by data returns to the CCG and direct knowledge of working with the practice teams. Practices were selected to include a wide range of sociodemographic and practice organisational characteristics. Each was offered a small honorarium to acknowledge their involvement and support data collection. Eight practices were initially recruited with two having to withdraw at separate points during the study (prior to qualitative data collection) due to staffing difficulties.

Recruited practices were already recording frailty status as part of routine care in people aged 65 or older, using the Clinical Frailty Scale [10] to validate the Electronic Frailty Index [11]. During the seven-month project 2,061 people with frailty over the age of 65 were involved in CSP. This equated to 67% of the practice populations over 65 years who had long term conditions (LTCs) and a verified frailty score (mostly mild or moderate).

Practices were asked to identify a falls champion who would lead in the practice and be a key contact to disseminate resources and actions to the relevant people.

### CSP frailty programme

To implement CSP, GP practices reorganise care pathways to include a comprehensive information gathering appointment. This is with a health care assistant (HCA) in which all screening, assessments, tests and tasks are completed for all of the conditions the person lives with or is at risk of developing. Routine test results and agenda setting prompts (patient preparation) are shared with the patient about 1 week before the care and support planning conversation with the nurse or GP and gives the person an opportunity to think about the questions, concerns and ideas that they wish to discuss with the health care professional during the conversation. The aim of the CSP conversation is to ensure that what matters most to the person (patient) is discussed alongside any professional concerns that have been identified.

The programme was developed by integrating additional elements for falls into the existing CSP process and providing bespoke training to share, and at times adapt, the process. An overview of the additional elements included in CSP to enable falls to be included can be found in S1 File.

#### CSP modifications

Practices were asked to add 3 questions at the information gathering appointment:

***In the last 12 months***:

1. *Have you had a fall including a slip or trip*?
2. *Have you had a blackout or found yourself on the floor*?
3. *Have you noticed any problems with your balance* (*e*.*g*. *whilst walking*, *standing up from a chair or dressing*?)

Those giving a positive answer to any question had their lying and standing blood pressure measured and were given a falls self-assessment leaflet by the HCA to prompt them to think about relevant self-management issues to discuss at the CSP conversation.

#### Preparation and the CSP conversation

For everyone with mild, moderate or severe frailty a generic prompt sheet for frailty was sent out prior to the CSP conversation in place of the previously used multiple LTC version. This enabled the issue of falls to be discussed in the context of either new findings e.g. hypotension/postural drop or general concerns around falls risk and frailty. Practitioners involved in CSP conversations were then trained to be able to support those who answered positively to the new questions around falls. A checklist based on the SPLATT acronym (Symptoms, (how did you feel?), Previous falls (how many?), Location (where were you?), Activity (what were you doing?), Time (of day eg. Day or night), and Trauma (any injuries?) [12] was offered to clinicians as a framework to identify potential underlying causes of falls. The teams were also made aware of local resources such as strength and balance classes and other interventions to support healthy ageing/prevention.

#### Support and training

Practices were supported to make modifications to IT templates to accommodate the additional activities/recording requirements and were provided with resources such as patient leaflets. Specific training in falls and frailty aimed to ensure that staff making the changes understood the rationale for the inclusion of the extra questions and that they were able to frame these in a way most likely to elicit a meaningful response.

Training sessions were approximately 90 minutes and in a variety of venues, some within and some external to and jointly with other practices. These were facilitated by 1–2 senior practitioners and included Year of Care team members on some occasions. The training included:

1. What is frailty and how it is identified?
2. Falls risk factors
3. Undertaking and interpreting lying/standing blood pressure
4. Having meaningful conversations about falls and frailty
5. Interventions to support healthy ageing/prevention
6. How to incorporate the new process into care and support planning

Each practice was provided with a resource folder during the training sessions. This contained details of the new process (laminated), relevant documentation, as well as information about local services, referral routes and signposting options to support healthy ageing. This was followed up with an electronic version.

Practice falls champions were contacted during the first 2–3 weeks of planned implementation to review progress. The local specialist falls coordinator offered practice visits to address any queries or concerns raised and 3 of the 8 practices accepted these.

### Qualitative evaluation

The evaluation team (TF, JS, MF & SH) were part of the overall project team that included also members whose roles were primarily focused on CSP implementation in the primary care context (HK, DD), oversight of CSP training and delivery (LO, SR) or overall conduct of the project (FS). All involved worked closely together and henceforth will be referred to collectively as ‘the project team’, except in describing data collection and analysis procedures, where reference to ‘the evaluation team’ (referring specifically to evaluation team members), is required.

#### Theoretical approach

The study was theoretically informed by Normalization Process Theory (NPT). NPT [13, 14] is a sociologically informed theory of how new interventions in (health and care) practice are implemented and become embedded as ‘normal’ practice. It focuses on how different groups of participants involved in the process of implementation *work together* to achieve implementation in relation to four key domains of activity: making sense of the practice change and gaining a shared understanding of the purpose and value of it and how it differs from previous practice (coherence), participation and sustained engagement in the activity (cognitive participation), successfully working together with the new practice within its setting (collective action) and reflecting on and appraising the impacts of the activity in ways that can be used to improve the process for those involved (reflexive monitoring).

#### Data collection

Group interviews and observations were undertaken across six general practices between November 2019 and February 2020, following the withdrawal of two practices prior to collecting these data.

*Observations*. In each study site, training sessions were observed by the evaluation team. They recorded observational notes which covered themes such as who the participants were, how the aims of the programme were explained and received by participants, any challenges or concerns raised, questions that were asked and the researchers’ own reflections. Training sessions were attended by a mix of staff members including GPs, practice managers, HCAs, nurses, a pharmacist and student nurses.

*Interviews*. Group interviews were conducted with staff involved in the programme following the training session, and after sufficient time had passed such that practice staff had experience of delivering the programme. The project lead organised this via the practice falls champion who then invited staff to participate in a group interview. Prior to group interviews participant information sheets, and consent forms were shared and the evaluators audio recorded the discussion.

A semi-structured interview guide loosely informed by NPT’s high-level concepts, which covered questions in relation to the training, potential impacts of training and processes of implementation of the programme, was used. Due to the nature of general practice there were at times some interruptions and staff who could not stay for the planned duration of the interview. On average the group interviews lasted 30 to 40 minutes.

#### Participants

Seven group interviews were conducted at six out of eight sites that were delivered training for the CSP programme. Original group number assignment (1–8) is retained in reporting of quotes. A total of 31 staff participated (n = 2–8 per group). Staff included practice nurses, health care assistants, nurses, and administrative staff.

#### Data management and analysis

Interviews were audio-recorded with participants’ consent, transcribed, and anonymised for analysis. Data were initially analysed collectively by the evaluation team, using thematic analysis [15]. Transcripts were initially read independently by at least two members of the evaluation team, with initial codes noted against data on the transcripts. In subsequent evaluation team analysis sessions (2–3 team members) interview transcripts were taken to identify key issues, discuss and agree coding labels to build an analytical map of key themes and connections between themes in the data. As each key theme was developed, it was tested against examples from observational data sources, and from different practices in the pilot. In this way, challenges to initial interpretations of data were actively sought out. This was an important part of the process of understanding variation in practices, and experiences at the different study sites, with reference to features of the practice contexts. Meetings of the wider project team towards the end of the project ensured that the qualitative analyses were informed by insights from all members of the team. Care has been taken in the reporting of findings to ensure that data is not attributable to individual participants and sites/GP practices. In reporting quotes, we refer to sources in relation to practice number and professional role only.

#### Ethics and informed consent

Formal ethical committee review was not undertaken, because the project was assessed as service development and evaluation, using the decision tool for categorisation of activity as research versus evaluation in the UK National Health Service (http://www.hra-decisiontools.org.uk/research/). This was further confirmed by North of England Commissioning Support 11/09/2019). However, written informed consent was still obtained from all study participants for their involvement in data collected for the evaluation. Before participating in the evaluation, participants received information sheets that met the general requirements of ethical approval committees, and they signed consent forms on the day of data collection, confirming willingness to take part.

## Results

**Fig 1:**
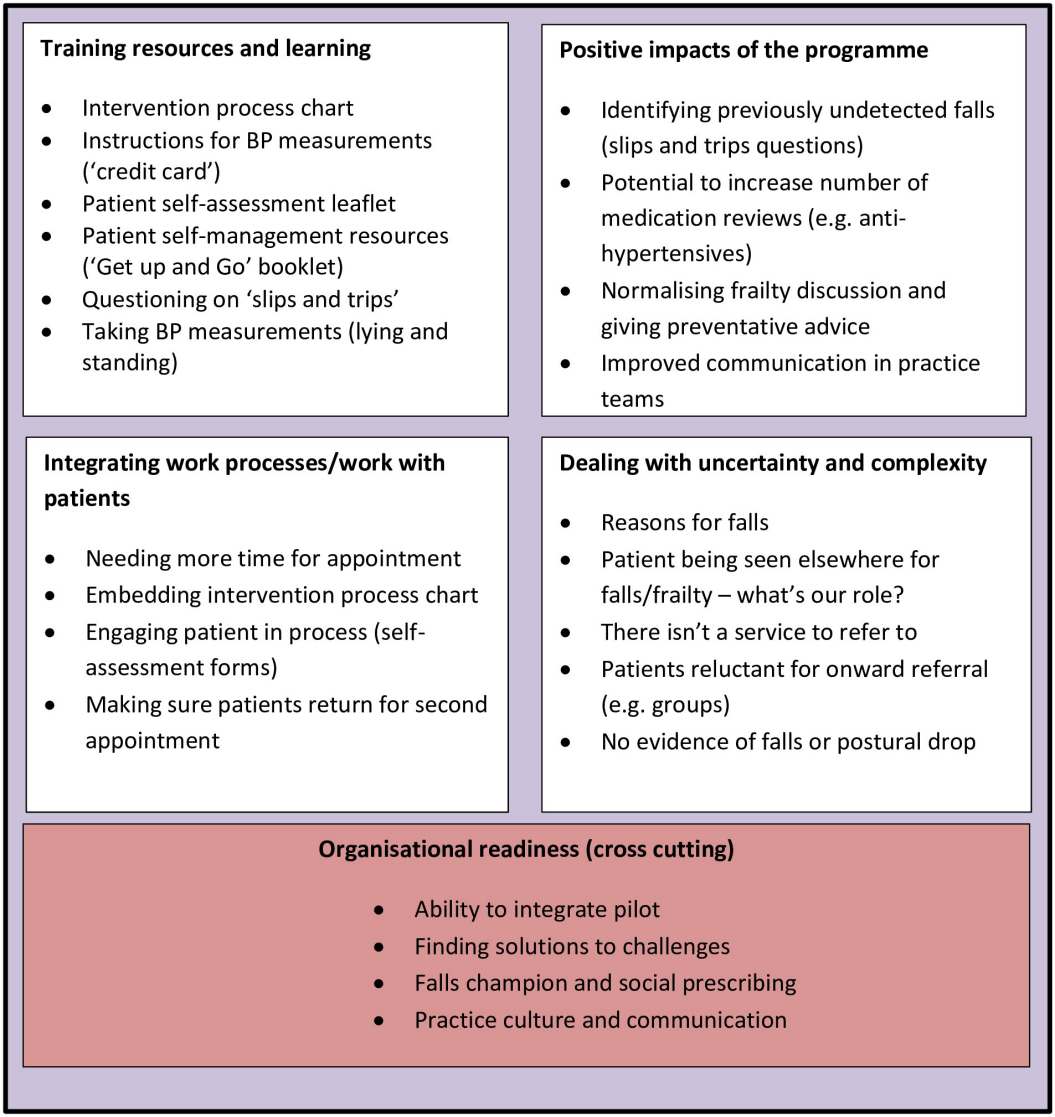
Overview of findings.

An overview of the findings is provided in Fig 1, and the discussion is organised according to the five key themes represented: Training resources and learning; Positive impacts of programme; Integrating work processes/work with patients; Dealing with uncertainty and complexity; and CSP readiness. Site readiness for CSP for frailty (to be referred to henceforth as ‘organisational readiness’) is a cross-cutting theme that runs through the other data themes; as such, findings in relation to the data themes are reported with reference to the observed readiness of the sites for the addition of falls to existing CSP. In relation to this latter theme on site ‘readiness’, we note that distinctions between sites that were ‘more’ or ‘less’ established in their CSP practices were made collectively by the project team, drawing on the qualitative data reported in this paper, including observations, and from other descriptive information about practices’ CSP service provision activity that was available to the team through steering group and other operational meetings.

### Training resources and learning

The observations showed that the training fulfilled its objectives and training facilitators seem to have judged the overall approach, breadth, and depth correctly. The content was well pitched and there was a sense of genuine learning during the sessions. The approach helped to overcome reluctance to implement the approach and increased buy-in.

As previously described, the initial support included resources intended to facilitate implementation and increase patients’ levels of active involvement in their CSP. Between practices, how these were used differed, ranging from participants never having seen any of them prior to training or not associating them with the programme, to some individual staff members/groups using resources but others not, to active engagement and integration of the resources into everyday workflow.

#### ‘Slips and trips’ questioning

The ‘*slips and trips’* questions were perceived as a key benefit from the training, as these gave participants the tools to ask patients more effectively about whether they’ve had a fall or were at risk of falling. Participants felt that ordinarily they would not have asked patients about falls or they would not have done it with the level of nuance the training had encouraged them to.

*Well yes because we wouldn’t have asked them if they’d had a fall*, *at all […]*. *We would have let them tell us they’d had a fall but we would never have asked them if they’d had a fall*.

*(Practice 8*, *nurse)*

*And I always remember […] at the training [they] said ‘be careful how you word it’ and she just said ‘just mention slips*, *trips and falls’ rather than saying ‘have you fallen*?*’ because they will instantly go ‘no’*. *So the first thing I say is ‘have you had any slips*, *trips or falls in the last 12 months*?*’*

*(Practice 8*, *nurse)*

The learning in this respect could be higher for the less organisationally ready practices where staff said they may have been less likely to ask patients about falls at all.

The slips and trips questions are seen to have raised awareness of frailty overall and equipped participants with a more nuanced understanding of falls and their causes, and more confidence.

*A lot of the people who fall*, *when you drill down you find it’s to do with something that might have been avoided*. *And it’s really trying to get in before that […] by asking those questions*, *to get people to think a bit more*.

*(Practice 2*, *nurse)*

#### Blood pressure measurements

An additional key component of training concerned the requirement for HCAs to take two blood pressure measurements if people answered positively to the slips and trips questions in order to assess a possible postural drop. For some, this required a change in flow of work at the information gathering appointment:

*I’m trying to get into the habit because you get in routines of how you do your reviews (others agree) and my review always started with the blood pressure but obviously my review needs to start with the question because if I’ve already done the blood pressure and then they answer the question I’m doing it again*.

*(Practice 8*, *HCA)*

Several participants said they had not known that it was possible to take a lying BP measurement by asking patients simply to elevate their legs onto a chair whilst sitting. Although trainers made it clear that guidance supported taking a lying measurement, leg elevation was seen as preferable to sitting alone. This was grasped as useful learning and less disruptive to the information gathering appointment. This should be recognised as a pragmatic compromise to the intended process and may require greater clarity in the future.

*I think if they can lie down*, *great*, *but this offers a different way of doing it which apparently is just as good*. *Because I didn’t know*! *I mean I’ve been a nurse for years and I didn’t know that*.

*(Practice 7*, *nurse)*

In several teams however, uncertainty about the number of BP measurements, the timing of this in the appointment, and whose role it was to take them, was evident:

*So basically*, *I’m doing the lying and standing BP*, *documenting it and then I think that’s me done (other participants in the group agree)*. *So I don’t know if I’m supposed to do any more or whether it’s up to whoever sees the patient next*.

*(Practice 2*, *HCA)*

In the practices with well-established CSP there were fewer questions about this step.

*Anyway*, *so all this is happening*, *it’s just that we have incorporated this into what we were already doing*. *So we do long term condition reviews anyway*, *we do Year of Care […]*. *So routinely they come in*, *they have their preparation appointment and then the results are sent out to them with their preparation paperwork*. *Which obviously*, *if appropriate the falls things are added into that*, *and then they come back and see ourselves for the second appointment*, *for the care and support planning appointment […]*. *So we’re not doing anything different*, *we’ve just added in the falls pilot to it*. *So [HCA] has been working very hard doing all the lying*, *standing BPs and asking the questions*!

*(Practice 1*, *practice manager)*

Particularly in reference to BP measurement, several participants expressed preference for the training to take place in their own practice. They felt that an in-situ demonstration of some of the required changes would facilitate implementation. Those teams less confident in CSP expressed that additional check-ins or the opportunity to ask for clarification, would be helpful. Practices who were more proactive in implementing the learning from the training, indicated that opportunities to share their experiences with other teams would be beneficial to further expansion of the approach.

### Positive impacts of the programme

Several benefits from having participated in the programme were reported. These included increased skills in identifying previously undetected falls by asking the slips and trips questions, the potential to increase the number of medication reviews being undertaken as a consequence, being more confident to discuss frailty with patients and giving preventative advice.

The training was felt to have led to greater awareness of falls: the slips and trips questions particularly, may help to detect otherwise missed falls. This increased awareness of frailty more generally was seen to normalise conversations about frailty, potentially improving patient experience/outcomes.

Participants reported they were picking up more people who were falling but may also be better equipped to determine root causes for falls, which may not always be associated with frailty but often have circumstantial reasons.

*The questions are good but I suspect the self-assessment is even better*, *because they go home and they chat and then their husband says ‘actually*, *that’s the third time you’ve tripped on that doormat*!*’*

*(Practice 2*, *nurse)*

There are also indications that this increased awareness and skill may lead to an increase in medication reviews and, ultimately, better outcomes for patients.

*I don’t know whether it’s from this [programme] but I’m more proactive at reducing people’s medication*, *especially their antihypertensive […]*.

(*Practice 4*)

Participants reported that they gained an overall increased confidence to talk to their patients about frailty, to pick up associated problems that may otherwise have gone unnoticed, and to suggest paths of action they might not have thought of.

*I suppose it highlights with the patient that it’s not normal*, *they don’t have to just put up with it*. *So… because a lot of them they will say ‘ah*, *that’s just me age’ but I suppose it just says ‘it doesn’t have to be like that*, *there’s something you can do’*.

*(Practice 4*, *nurse)*

*But as well it made me think about some of the other things that I wouldn’t always think about*. *So opticians*, *for example*, *obvious things but it gave me a bit of a checklist almost of things to think about*. *Which has been really useful*. *And also*, *when we do home visits now*, *the preparation for the home visits is different […] it’s just kind of helped with doing the visits and having the information*.

*(Practice 1*, *nurse)*

In some instances, the learning from the training has improved communication in the practice team and encouraged HCPs to take a more active overview of a patient’s care needs/trajectory.

*Frailty and falls*, *before doing the training*, *I wasn’t really sure what to do with it*. *And I think just being able to speak to people about that*. *But also […] just being able to speak to the GPs about maybe changing their meds slightly if they are on anti-hypertensives*. *But I suppose also talking to them about staying hydrated and things like that*. *So yeah*, *I guess that’s kind of where I’m at with all of it*.

*(Practice 2*, *nurse)*

### Integrating work processes/work with patients

Despite the learning gained, not all staff found it easy to adjust their workflow in a way that accommodated the intervention in accordance with the delivery process chart.

Practices who were newer to CSP reported finding it more difficult to keep sight of the intervention and to integrate it into their workflow.

*“For me personally*, *because I’m dealing with everything else*, *it’s not the top of my priority list*, *and I’m sorry about that but you know […] there’s no reason it shouldn’t be*, *but it’s just because I’m not used to it*, *you know what I mean*, *it’s something quite new*.

*(Practice 8*, *HCA)*

In the practices where CSP was less established it might have been considered an unnecessary burden or that there was simply not enough time during the appointment to fit all the required elements in.

*Yeah we haven’t seen those [resources] and we haven’t got sufficient time to do a standing and lying blood pressure either*, *they only get a normal BP done*.

*(Practice 2*, *HCA)*

If the programme was to be sustainable many participants felt there needed to be some flexibility in appointment length.

Some of the challenges, however, may have been related to staff members’ own levels of confidence in spotting the necessary problems, and in how to address them and who to refer to. Practices’ own processes regarding communication, appointments, and workflow varied in terms of alignment with delivery of the programme. The more ‘CSP-ready’ practices seemed to have found it easier to talk to colleagues.

*If I’m concerned about somebody then if they’ve got a second appointment for Year of Care then I put it in the consultation for the second appointment and then these two ladies will see it when they see the patient next*. *If they haven’t got a second appointment and I’m concerned*, *then I’ll get in touch with the GP and send a (task) to a GP*.

*(Practice 2*, *HCA)*

Integrating the programme into the existing workflow was also seen to be affected by patient engagement. Some may simply refuse to engage in the process (e.g. second appointment, engaging with resources, accepting referrals).

*I liked the motivation [of the training team]*, *we’re really motivated but then I think sometimes we feel a bit disheartened when we come back and try do it in General Practice and we’ve got an elderly person sitting in front of us saying ‘I’m not going to go to an exercise class*, *howay pet*!*’*.

*(Practice 5*, *nurse)*

For some participants, approaching the programme as a series of appointments across which to build engagement, was a useful strategy.

*It’s raising awareness in the first appointment with the patients so they can maybe focus on that and focus thoughts on that and bring it with the results*. *It all becomes incorporated then in the whole thing that there is a specific focus for them about falls*. *Through conversation and having the first appointment and having the lying and standing BP done and why we’re doing it*. *So you’re giving them information*, *raising awareness*, *ready for the second appointment*.

*(Practice 1*, *nurse)*

Making sure that patients come back for their second appointment was a potential challenge: however practices reported their own strategies to address this.

Participants also reported very practical ways in which they worked to support integration of the programme into everyday workflow. These included creating and posting flow diagrams of the process in every consultation room, and in one practice, integrating the slips and trips questions and BP measurements into their own computer templates to ensure they were remembered, and that the information would be captured on the system.

*We’ve just designed this ourselves so that it doesn’t get forgotten*. *So we know that anyone who comes in for Year of Care*, *if they are over 65*, *it’s the first thing that comes up when the health care assistants go on the template so that we know it gets done*. *And then they fill that in*, *because there’s three questions that they ask*, *so if they’ve had a fall then they would continue with the rest of it and then they would do a lying-standing blood pressure*.

*(Practice 7*, *nurse)*

### Dealing with uncertainty and additional complexity

Despite picking up more falls in conversation by using the slips and trips questions, participants were not always sure how to proceed when falls were caused by reasons clearly not related to frailty (e.g. tripping over the rug) or if patients were already seen by other services such as specialist teams or other support.

The intervention process chart did not make it sufficiently clear for HCPs, what to do with those patients who do not sit neatly within the process.

*For instance*, *this morning I had somebody in and she said that she was a little bit dizzy but she’s had no falls*. *So straight away then my line of questioning changed a little bit different direction and what we ended up coming up with was*, *because her blood pressure was slightly on the low*, *we’d do more water intake and stuff like that*, *you know*. *And pop back and see us if there were any problems*. *Her daughter was there*, *which was well on board*, *she says ‘I’m pleased you’re seeing this’ because what you’re doing is reinforcing everything I have been saying*, *you know*. *But she’d had no falls as such but even the dizziness*, *that made me think ‘hm*, *hold on’ you know*, *and took a different turn you know*. *So on the frailty’s (scoreboard) it sort of went up a little bit but it was noted ‘no falls’ but thinking this blood pressure might be… you know*, *so*.

*(Practice 2*, *HCA)*

Similarly a recurring question participants had was what to do with those patients who were already seen by other, specialist, services not least because it was considered a wasted appointment by some.

*One of [my patients] was already under Falls*, *the Falls Clinic*. *So I was a bit confused as to what to do with them*, *because they’re already technically under the team as it is*.

*(Practice 8*, *HCA)*

*And then there was another one*, *he answered positive to the slips and trips but they were unstable with their mobility*, *they were already on crutches and they were already under orthopaedics and things as well*. *So when they were already under people for things and the falls were contributing to something*, *like it’s from our end*, *what to do with them*?

*(Practice 8*, *nurse)*

There was also uncertainty around what to do if there isn’t a service to refer a patient to, or if the patient does not want to engage, and what should be done if there is evidence of frailty but no evidence of falls or a postural drop. Staff recognised both the value of referrals to services such as ‘strength and balance’ classes, and the challenges in engaging patients’ participation in them:

*I can think of a couple of patients where we’ve possibly picked up on something and then I had a patient who fell and she broke her wrist and going to Staying Steady brought her confidence back because she was worried about driving again*. *So going to do that*, *her confidence was better and then being able to drive didn’t isolate herself and her husband*.

*(Practice 2*, *nurse)*

*outcomes have changed*, *whether it’s the medication or whether they’ve agreed to go to Staying Steady*. *But I still find that a lot of them are quite dismissive of Staying Steady and I just get frustrated by how little we’ve possibly done because our patients like to (come to this building don’t they [name omitted]*!*) ((some laughter from group)) and they don’t like to go anywhere else*! *It doesn’t matter if it’s two doors that way or five doors that way*, *they like to come here*!

*(Practice 2*, *nurse)*

### Organisational readiness (cross cutting theme)

As indicated in reporting thus far, a key factor in the successful and consistent implementation and the degree to which practices embraced the programme is how established the CSP was and how willing and able they were to add in the elements relating to falls (organisational readiness). Existing approaches to this within the practices mattered:

*Yeah*, *we’ve always tried to do what we can when we see people for their reviews*. *Whether that’s referring them to Social Services or referring them here*, *there or everywhere or if they need some extra support with whatever*. *So we’ve always done that when we see them*. *And then for the last couple of years when the frailty’s been*, *sort of on the agenda and we’ve had to start scoring everybody*, *it’s kind of stepped up again*. *And then obviously just adding this in has just made a little bit more comprehensive I suppose*.

*(Practice 4*, *nurse)*

In practices where CSP is itself more established, the feedback demonstrated an engagement with and understanding of the training that reflects the key changes/additions to the YOC process: asking the slips and trips questions, taking an additional BP measurement and handing out appropriate resources/referring the patient to the most appropriate health care professional or service, either in-house or external. In these practices the willingness, ability and confidence to do this were high as teams could see the benefits of doing so and they did not feel that the intervention asked them to do anything too onerous.

*We do the frailty assessments anyway*, *it’s something that’s in our minds anyway*. *It’s something that we ask about when we see them*, *even if not at the initial appointment then at the second appointment we ask about that kind of thing anyway*. *So this has just made it easier really because you’re asking about it at the initial appointment and then you’re already putting things in place to be able to review things*. *Whereas before*, *we might have asked about it at the second appointment and then they might have needed to come back in for lying and standing BP and for further assessment and things*. *So it’s making it more a part of that second appointment by having it already done*.

*(Practice 1*, *nurse)*

Likewise, the increased focus of the programme on elements of social prescribing aligned well with the existing practices of some of the practices:

*Normally*, *like I say*, *we use the care navigators and the frailty nurse or the community matron if we think that we need support like that*. *Yeah*, *I suppose just signposting them to all the services out there*. *There’s things like the Life Programme*, *signposting that quite a bit and the Older Person’s Assembly*, *they’ve got lots of activities*, *so I try to give them options*, *not just Staying Steady for like…*

*(Practice 4*, *nurse)*

In summary, key challenges and enablers to implementing the intervention were observed and reported, according to practice levels of readiness for implementing the programme. Some challenges were shared across all practices regardless of levels of CSP-readiness, in particular uncertainty around those who did not easily fit into the frail category but had reported slips, trips or falls caused by unrelated issues (e.g. tripped over carpet) and/or those patients who were already seen by other specialists (e.g. falls clinic) or elsewhere. The data suggest that the more established in CSP and frailty a practice was, the more willing and proactive they were at finding solutions to some of these challenges.

## Discussion

This evaluation identified key experiences of and factors affecting the identification of falls risk as part of the routine management of people living with frailty and long-term conditions, within UK general practice, using care and support planning (CSP).

The project included people with mild to moderate frailty which is the group with most to gain from prevention and intervention [16]. To date the demonstration that falls risk can be ameliorated, most notably by interventions to improve strength and balance, has involved irregular recall of at-risk individuals for time limited projects focussed only on falls [4]. CSP was chosen for this project because it potentially offers a method of routinely and proactively identifying those at risk from falls and creates an opportunity to discuss preventative interventions early and be discussed annually [2].

Using a CSP approach has additional benefits for the person and the practice. For the person it ‘normalises’ the concept of regular reflection and self-reflection on health issues which is a central ethos of CSP, engaging people in their health and enhancing their knowledge, skills and confidence to self-manage and problem solve issues as they arise. CSP also enables falls risk to be assessed and discussed alongside other amenable issues of ageing, within a holistic framework focussed on ‘what matters’ to each person in living their life. It enables frailty and falls to be included within existing care pathways without creating new services or a significant increase in costs or care burden for the practice as well as the patient.

Our qualitative evaluation revealed additional, unexpected benefits for staff development. By highlighting all the health issues related to frailty within CSP those involved reported enhanced interest, understanding, knowledge and confidence in talking about frailty and falls in general. Staff welcomed this; a number described it as a worthwhile extension of what they are doing both within CSP and with potential for their overall work within the practice.

This feasibility programme was not designed to assess health related outcomes and provides no evidence on whether this routine, holistic approach to the ageing population would lead to health benefit for individuals. Routine CSP needs to be seen as part of a more complex intervention in which the outputs of the conversation are linked to personalised, community support to improve strength and balance or address other risk factors identified via CSP (social prescribing). Attempts to link with such activities, which had not been specifically included within this project, proved difficult. However, this evaluation identified key issues which would need to be addressed when designing such an approach in the future. Interviewees were not always confident in how to discuss these activities with patients nor make the links. They reported that strength and balance classes were not widely available and were unclear about referral mechanisms for this and other relevant ‘social prescribing’ activities despite its current high profile. Work by the CCG and Primary Care Networks (PCNs) to develop these services will need to focus on embedding better understanding and developing relevant skills among practice teams as well as ensuring the community activities are available and easily accessible.

The initial training programme to support implementation proved essential and received positive feedback on content and style of delivery. The evaluation identified key practical issues including the importance of timing and venue, collating the suggestions for improvements to the training which have now been included alongside other recommendations for support.

Regarding the implementation and integration of falls within CSP, from a Normalisation Process Theory (NPT) perspective ‘coherence’ was relatively high; staff generally recognised the value of incorporating falls and frailty routinely and its positive impact on patient care. This helped to increase confidence in the enhanced CSP conversation. The ease with which the new elements were included into the process, or the ‘workability’ of the programme in practice (as described in NPT’s concept of collective action), was linked to the degree practices had previously embraced and implemented the YOC model and a systematic approach to frailty. In those where the model was well embedded, staff were more positive of the approach, appreciated its relevance within CSP and were able to engage more fully across the different components (‘cognitive participation’). Clarity around roles and ascribed tasks also seemed to support more successful implementation. This is valuable learning for further implementation.

A key observation was the need for those involved in all forms of support to recognise that one size does not fit all for practice teams. The importance of each training element and the request for additional support varied from practice to practice. Two broad groups of practices were apparent. One group were confident in their approach to CSP and previous work with frailty and found implementation of change and new work around falls relatively easy. For others it was less so. Some of the differences involved specific components of CSP and frailty. Others seemed to relate to broader practice characteristics, routine procedures and the whole team approach to introducing new ways of working.

All these features influenced the effectiveness of initial training, which was broadly sufficient for ‘ready’ practices, and the request for additional in-practice and ‘just in time’ top ups, which proved essential for the more ‘hesitant’. The evaluation found that opportunities to meet together for both groups, although difficult to organise and time consuming, offered additional value providing a forum to share experiences, learn from each other and work out solutions to practical problems. Going forward the project recommends that training and support must be flexible, building in options and tailored to practice need which might involve specific work to identify this.

The evaluation generated other important learning for wider implementation and spread to other sites. A key message is that incorporating the specific elements related to falls and frailty was only possible within the time frame because practices were already experienced at delivering CSP for those with multiple LTCs.

The role of a practice ‘falls champion’ was proposed at the start as a key, named member of the team who might volunteer to act as a conduit between the practice staff and project team; to support practice implementation, introduce resources and highlight any issues. The evaluation did not set out to investigate the role of the champion in great detail, but feedback from the focus groups suggests this was not as successful as intended. Despite these champions demonstrating that they were keen and enthusiastic, they did not always have the authority or time to influence change in practice. For instance, some participants expressed that they had not been provided with the expected information and resources prior to training and this would need to be checked in the future. Champions were not offered any additional training than that received by all staff, so on reflection, it may be possible that suboptimal levels of confidence and competence in dealing with CSP for a frail population may have impacted on their potential effectiveness as champions within their teams. Such a ‘champion’ role, as a focus for a practice activity in a specific subject as well as for CSP in general, has been helpful in previously successful implementations of CSP that the project team have been involved in, and could be important in embedding understanding of falls and frailty in the future. A relatively recent large review of literature on the role of champions in healthcare implementation found that individual studies consistently reported champions to have had positive influences on implementation [17]. Their review however, highlights much diversity in the roles of champions in individual studies and a breadth of factors that have been reported to influence their effectiveness in implementation efforts. The barriers to and success criteria for such a role in relation to CSP, particularly for frailty, need further investigation.

### Strengths and limitations

The evaluation of the implementation of CSP in routine service provision is a key strength. Together, the practices represented a wide range of demographics, social deprivation, prevalence of frailty, and different organisational processes of care delivery. The close working of the multidisciplinary team (researchers, service providers and expert providers in CSP) enabled comprehensive scrutiny of the findings and translation of findings into practical recommendations. The qualitative methodology, including applying NPT as a theoretical framework to focus data collection and analysis, further facilitated in-depth exploration of the implementation and integration process, identifying success criteria for CSP more generally and how to increase the effectiveness of implementation.

Implementation issues in some of the sites (e.g. staffing turnover, release for training, logistics), and differences in success in resolving these challenges, meant that some sites did not achieve a level of activity to generate as much learning as hoped in relation to staff experiences of delivering falls focused CSP to patients. Assessing the quality of the interactions between patients and practitioners in the different elements of the CSP process, was also not possible in this project. The diversity of experiences of implementation however, did contribute valuable learning about the implementation process and challenges that need to be further addressed.

Some limitations regarding the participant sample are also noted. The focus on those already receiving CSP excluded those over 65 years without core LTCs, with or without frailty, who may still be at risk of falls. Including these would increase the broader population that may benefit from the intervention but increase the costs. Take-up of CSP is also around two thirds of those invited. Further understanding of the overlap between LTCs, frailty and CSP, along with participation factors, is needed.

## Conclusion

Care and Support Planning services designed to target frailty and falls is feasible and can successfully be delivered in the primary care setting. As a complex intervention, implementation was not without challenges and some practices found that they were able to implement frailty assessment more easily as part of CSP, than were others. However, practices that were delivering CSP for long term conditions, felt that the adapted CSP process for frailty was a logical extension to their current work. Not only did this prove possible but highlighted the potential for this approach to increase and embed greater interest and understanding of ageing, frailty and falls in their day-to-day work as a component of staff development.

## Supporting information

S1 File (DOCX)
