# Supplementary material for: Using care and support planning to implement routine falls prevention and management for people living with frailty: A qualitative evaluation
Source: PLoS One. 2022 Oct 11;17(10):e0275974. doi: 10.1371/journal.pone.0275974 (PMC9553036; doi:10.1371/journal.pone.0275974)
Supplement: S1 File — (DOCX) [file pone.0275974.s001.docx]

**Supplementary File 1**
